# Emergence of sense of body ownership but not agency during virtual tool-use training is associated with an altered body schema

**DOI:** 10.1007/s00221-023-06644-3

**Published:** 2023-06-12

**Authors:** Amir Jahanian Najafabadi, Dennis Küster, Felix Putze, Ben Godde

**Affiliations:** 1 Department of Cognitive Neuroscience, Bielefeld University, 33501 Bielefeld, Germany; 2 School of Business, Social and Decision Sciences, Constructor University Bremen, 28759 Bremen, Germany; 3 Department of Computer Science, University of Bremen, 28359 Bremen, Germany

**Keywords:** Virtual tool-use, Forearm body schema, Arm representation, Sense of ownership and agency

## Abstract

In this study we examined if training with a virtual tool in augmented reality (AR) affects the emergence of ownership and agency over the tool and whether this relates to changes in body schema (BS). 34 young adults learned controlling a virtual gripper to grasp a virtual object. In the visuo-tactile (VT) but not the vision-only (V) condition, vibro-tactile feedback was applied to the palm, thumb and index fingers through a CyberTouch II glove when the tool touched the object. Changes in the forearm BS were assessed with a tactile distance judgement task (TDJ) where participants judged distances between two tactile stimuli applied to their right forearm either in proximodistal or mediolateral orientation. Participants further rated their perceived ownership and agency after training. TDJ estimation errors were reduced after training for proximodistal orientations, suggesting that stimuli oriented along the arm axis were perceived as closer together. Higher ratings for ownership were associated with increasing performance level and more BS plasticity, i.e., stronger reduction in TDJ estimation error, and after training in the VT as compared to the V feedback condition, respectively. Agency over the tool was achieved independent of BS plasticity. We conclude that the emergence of a sense of ownership but not agency depends on performance level and the integration of the virtual tool into the arm representation.

**Supplementary Information:**

The online version contains supplementary material available at 10.1007/s00221-023-06644-3.

## Introduction

Tool-use, i.e., the ability to handle an object (the “tool”) to reach, manipulate or grasp another object is one of the most important hallmark skills of the human species (Miller et al. 2017; Cardinali et al. 2012), and can help us to overcome the limitations of our bodies. Researchers used various experimental approaches to understand how short- and long-term tool-use in humans modifies the body representation (BR) and body schema (BS) (Martel et al. 2019; Day et al. 2017, McCormack et al. 2011; Miller et al. 2014), and how a sense of body ownership and a sense of agency (in the following referred to as ‘ownership’ and ‘agency’), and feelings of control over the tool and its movements emerge (Nava et al. 2018; Jung and Hughes 2016). In this study we transferred and adapted a tool-use paradigm introduced by Miller et al. (2014) into an augmented reality (AR) environment and explored whether effects on BS would be similar as those found by Miller et al. (2014) in a real-world setting. Further, we were interested in how the emergence of ownership and agency over the virtual tool would depend on induced changes in BS and how this association would depend on the type of feedback provided (vision-only or visuo-tactile feedback).

### Ownership and agency

Ownership refers to body parts being perceived as belonging to one’s own body and is the sense that “I am the one that is going to experience, e.g., when one’s body is moving regardless of whether voluntarily or involuntarily” (Gallagher 2000, p15). Agency is the feeling that actions or events are produced by one's own body, and that the agent is the cause of its own action (Gallagher 2000, 2018).

Ownership results from the integration of somatosensory and vestibular inputs, which correlates with activity of certain brain structures corresponding to sensory processing and motor control (Tsakiris and Haggard 2005). Cardinali et al (2012) & de Vignemont (2007) have further proposed that the body schema (BS) becomes a source of ownership because it constitutes the spatial content of the bodily sensations that localise bodily properties within the BS. As demonstrated by Ma and Hommel (2015), non-corporeal objects can likewise be perceived as parts of one’s own body. This provides evidence against the idea that ownership relies on pre-existing, temporally stable top–down body models, thus supporting bottom-up approaches (Ma & Hommel 2015). In further support of this notion, D’Angelo et al. (2018) revealed a significant modulation of the sensorimotor representation of the arm by use of a virtual hand that moved synchronously with their own hand movements. They further revealed significant effects of ownership and agency on the BS during virtual hand training.

In addition, ownership and agency are subject to multimodal integration like the internal models underlying motor control and can be experimentally manipulated (Grechuta et al. 2017; Serino et al. 2013; Petkova et al. 2011; for reviews on how ownership and agency are shaped by multimodal inputs, cf Blanke 2012; Noel et al. 2018; Tsakiris et al. 2007; Wen and Imamizu 2022). However, little is known about whether sensorimotor BS plasticity mediates ownership and agency in virtual tool-use experiments (Rubo and Gamer 2019; Cardinali et al. 2021).

### Tool-use alters the body schema

According to the dyadic model (for review: Cardinali et al. 2011; Head and Holmes 1911), two distinct subcomponents constitute the body representation: body image (BI) and body schema (BS; de Vignemont 2010; Dijkerman and de Haan 2007). The BI is a perceptual conscious representation of the body (Cardinali et al. 2012), and is involved in body perception, body affect (Cardinali et al. 2012; de Vignemont 2010; Gallagher 2005), and body concept (Segura-Valverde et al. 2017). The BS is seen as an unconscious sensorimotor representation of the body that is used for action planning and execution of movements (Martel et al. 2016; d’Angelo et al. 2018). In contrast to the BI which is seen as a stable representation of body shape and size (Cardinali et al. 2009a, b), the BS has been defined as a more short-term representation (Cardinali et al. 2012). It flexibly updates with every change in the state of the body, e.g., due to growth and body lengthening accompanying maturation—or as an effect of tool-use (Cardinali et al. 2012; Cardinali et al. 2009a, b).

Indeed, studies on neuroplasticity after tool-use suggest that the BS can be extended to incorporate tools, and other objects we manipulate in our daily life, into the representation of our body (Iriki et al. 1996; Romano et al. 2018). Tool-use dependent alterations of cortical activity patterns were also reported for the left intraparietal sulcus (IPL; Tomasino et al. 2012), and the posterior parietal cortex (PPC; Inoue et al. 2001).

Behavioural paradigms that have been leveraged to explore how tool-use affects the BS include tactile localization, distance estimation and motor control (Cardinali et al. 2009, 2012). Even 10 min of training with mechanical tools for arm extension and body position made participants experience slower arm movement in a reach-to-grasp task, which could be considered as stretching the arm relative to its central representation (Cardinali et al. 2012). Testing the perception of tactile distance has previously been employed to investigate somatosensory processing and BS plasticity (Miller et al. 2014, 2017). While participants usually underestimate the distance between two tactile stimuli presented to the skin, previous research revealed some asymmetry with perceived smaller distances along the proximodistal orientation compared to the mediolateral direction in tactile distance perception tasks with the arm or hand (Calzolari et al. 2017; Longo et al. 2010). This perceptual asymmetry might be related to anisotropies in the shape and organisation of tactile receptive fields (RFs) along the hand and forearm surface (Canzoneri et al. 2013; Longo and Haggard 2011). Previous studies suggested, for several body parts, that the perception of tactile distance between two points on the skin is strongly linked with the BS (Longo 2020; de Vignemont et al. 2005). Therefore, tactile distance judgement (TDJ) tasks may be valuable paradigms to investigate whether the BS of the hand, the forearm, or other limbs is altered after tool-use training (Sun and Tang 2019; Miller et al. 2014, 2017).

Miller et al. (2014) used a TDJ task where participants had to judge whether the distance between two stimuli presented in different orientations (proximodistal and mediolateral) on the skin were shorter or longer than a reference presented on the forehead. In these studies, the perceived distance between two tactile points on the forearm in the proximodistal orientation was furthermore significantly reduced after tool-use training. Results also revealed that representational plasticity was different on the hand and arm when manipulating the morphological similarity between the tool and the respective effector (irrespective of the function/goal of the tool for grasping and moving the objects). Training to use a hand-like tool and an arm-like tool (mechanical grabber) thus appeared to lead to alterations in the perception of the size of the hand or the arm, respectively (Miller et al. 2014). Jovanov et al. (2015) also spotted specificity of BS plasticity to the trained body part. Interestingly, Baccarini et al. (2014) revealed that tool-use imagery is enough to induce changes in the representation of the arm after participants performed imagery tasks with either a tool enlarging their arm length or with their hand alone as a control. In addition, according to the work by Miller et al. (2014) and Cardinali et al. (2016), the specificity of BS plasticity corresponding with the shape of the tool indicated transferability from hand-shaped tools towards real hand representations, altered grasping actions, and kinematics of the arm. Further, it seems the tool must possess functional significance to influence the neural representation as expected (Sposito et al. 2012).

### Tool-use in virtual and augmented reality

Immersive virtual reality (VR) can provide rich multisensory experiences. VR in combination with vibro-tactile feedback provided by so-called ‘cybergloves’ are gaining attention in investigations of tool-use training effects (Rubo and Gamer 2019). Previous research suggests that experience in VR affects both ownership and agency (Kong et al. 2017). VR has thus been used for stimulating body ownership illusions (BOIs), i.e., the illusory sense of ownership over a simulated body, in a very operable way (Braun et al. 2018). It was also confirmed that the integration of visuo-tactile stimulation together with one's own body movement in a virtual environment induces high feeling of agency over the virtual limb (Franck et al. 2001). In the same vein, previous work has consistently reported a positive impact of integrating visual and sensorimotor haptic feedback on task performance compared to visual feedback only (Prewett et al. 2012). Finally, Kong et al. (2017) used two avatars that performed goal-directed actions in a VR environment. Depending on their experimental paradigms, their study revealed that enhanced agency over a virtual avatar could emerge in VR. These authors also suggested that sensorimotor experience with a visible avatar standing in front of the virtual desk is critical for inducing misattribution of agency to an avatar. Furthermore, it was indicated that people tend to attribute the avatar hand’s action to themselves after controlling the avatar hand for a brief period. In comparison to VR, AR is a more recent technology that has yet to receive the same degree of attention in research on BOIs. AR generally provides greater feelings of presence and reality of judgement for the user than VR. For the purposes of the present study, AR can be used to control an interactive task, while still allowing the user to see their real hand. In comparison with VR training, AR thus enables participants to reach and grasp the object, and to consider other critical experimental variables during the tool-use training such as the size and shape of the object. Further, AR can more easily incorporate different aspects of the real physical environment than VR, which is based on an entirely artificial virtual environment (Lenggenhager et al. 2007; Juan et al. 2005).

### The present work

In this study, we transferred the tool-use paradigm introduced by Miller et al. (2014) into an AR environment. Participants had to grasp a virtual object with a virtual gripper attached to their own real hand. They were instructed to carefully touch the object with the central part of the gripper’s head but not with either of its protruding sides. This task requires precise control over the gripper and if one would assume some adhesive force between the surfaces, then the object might be ready for being virtually lifted.

After training, the participants answered questionnaires about their subjective feelings of ownership and agency over the virtual tool. To elucidate the role of visual and tactile feedback for the emergence of ownership and agency, participants were trained in two different conditions. In one condition, participants received only visual feedback where they saw the virtual object and virtual gripper in AR but also their real hand and the table. In the other condition, when (incorrectly) touching the object at either side, vibro-tactile feedback was applied to the thumb (left) or index finger (right) through a CyberTouch II glove, and when (correctly) touching the front of the object with the tool, vibrations were generated at the palm.

To test whether the training with the virtual tool led to a change in the arm representation in the BS, we used a TDJ task which required participants to estimate the distance between two tactile stimuli on the forearm oriented either along the arm axis in proximodistal orientation or orthogonal to it in mediolateral orientation.

We expected the training with the virtual tool to lead to a change in the arm representation in the BS, indicated by altered perceived distances between two tactile stimuli. Following Miller et al. (2014, 2017), we hypothesised that the perceived distance between two tactile points on the forearm in the proximodistal orientation, but not in the mediolateral orientation, should be reduced after tool-use training, indicating an integration of the virtual tool into the existing BS. We further expected that the amount of such changes in the BS should be associated with the emergence of ownership and agency over the virtual tool. In fact, these experiences might be comparatively salient in AR, due to their novelty in comparison with more familiar physical tools, but might depend on the type of feedback. We therefore predicted that combined visual and tactile feedback, as in real world settings, would be more effective than visual feedback alone.

## Methods and procedures

### Participants

We recruited thirty-seven right-handed healthy participants at the University of Bremen and Constructor University (Formerly known as Jacobs University Bremen). Previous studies reporting effects of tool-use on BS and BI usually had smaller sample sizes between 10 and 20 participants, including Miller et al. (2014). Because replications usually require larger sample sizes, we aimed at doubling this sample size. Although our design is different with respect to the AR method used, we therefore assume that our sample size should be sufficient. Participants were compensated with 10 Euros per hour. They possessed normal or corrected-to-normal vision, provided informed consent (participation and publication), and were naïve to the experimental hypothesis. Data of three participants had to be discarded due to the technical issues and sickness, and thus incompleteness of datasets, yielding a total of 34 participants (15 males, 19 females; *M*_age_: 23.64, *SD*: 7.07). All procedures were approved by the Ethics Committee of the University of Bremen and were in accordance with the principles of the Declaration of Helsinki.

### Study design

**Fig. 1:**
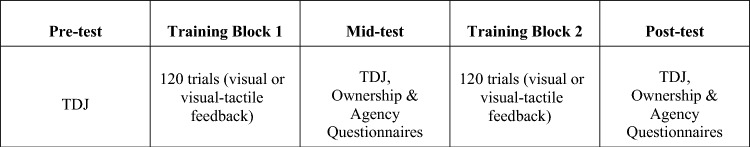
Experimental design

Participants underwent a virtual tool-use training in AR in training blocks with and without vibro-tactile feedback as described in the training section below. The Purdue Pegboard test was used at pre-test to measure unimanual and bimanual finger and hand dexterity of participants. BS was assessed by the TDJ (cf., Miller et al. 2014, 2017) and a tactile localization task (TLT). TDJ and TLT were conducted before training (pre-test), after the training block with the first feedback condition (mid-test) and after the training block under the other feedback condition (post-test). Both ownership and agency for the virtual tool were assessed with questionnaires at mid-test and post-test. Electroencephalography was obtained to record resting-state EEG patterns at all three time points and task-related EEG during both training blocks (cf., Fig. 1) and somatosensory event-related potentials were obtained during the TLT. As the focus of this paper is on changes of the BS and the question of whether the predicted changes in the BS were correlated with the emergence of ownership and agency over the virtual tool, we will report only findings from the TDJ and the ownership and agency assessments. Additionally, behavioural data as an indication of participant’ performance level during virtual tool-use training will be used in our model.

### Virtual tool-use training in AR

**Fig. 2:**
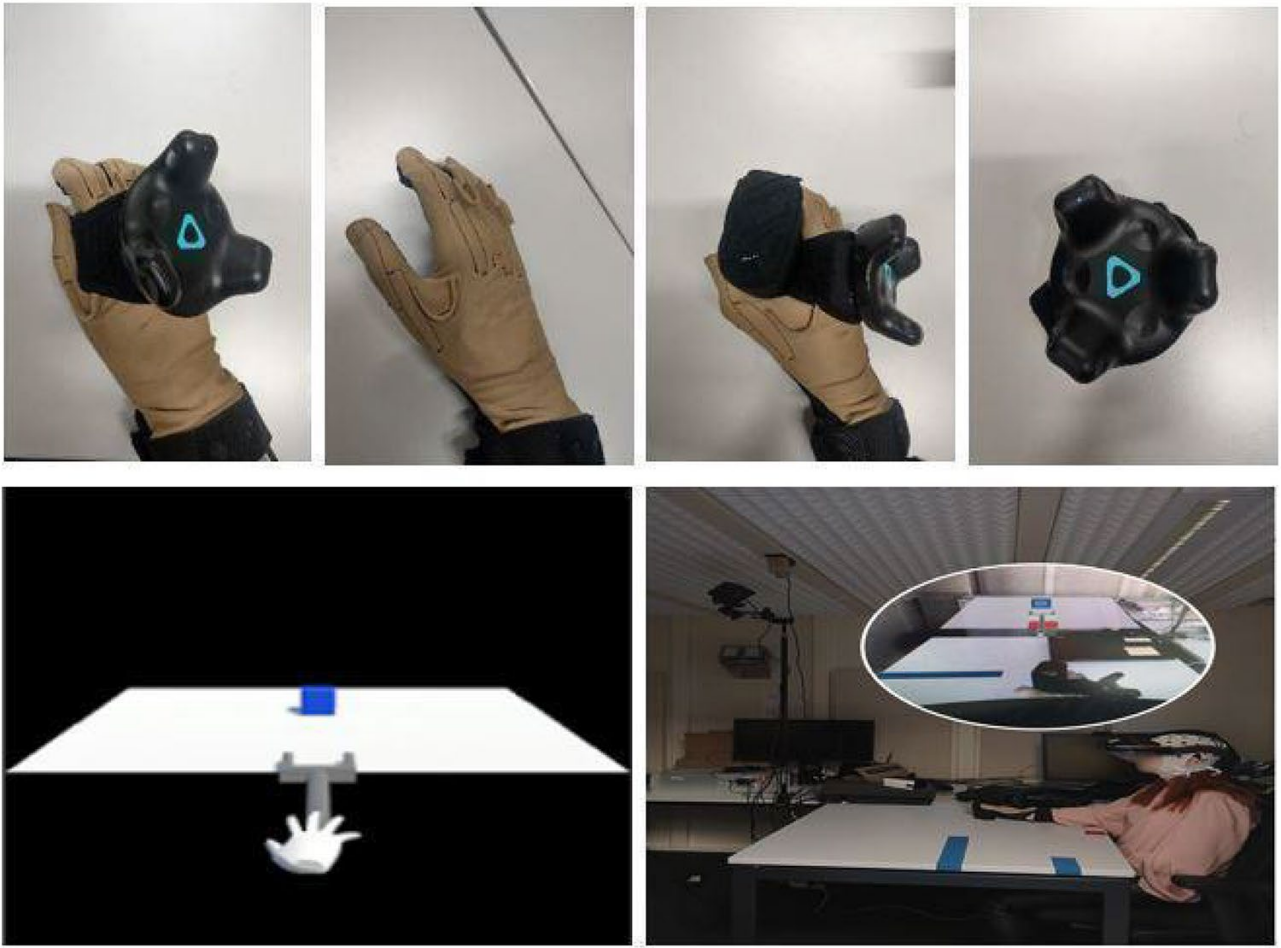
Experimental setup for tool-use training. Top row: CyberTouch-II and Wireless HTC Vive Tracker. Bottom row: Scene view

Participants sat in front of a white table, wearing a Meta2 AR headset (www.metavision.com), which included earphones for receiving verbal instructions. A wireless HTC Vive Tracker 2.0 model KLIM was attached to the back of their right hand. Next, participants donned a special glove (CyberTouch-II, CyberGlove System Inc., 2157 O'Toole Ave, San Jose, USA; cf., Fig. 2) on their right hand. The CyberTouch-II provides fine-grained vibro-tactile feedback on the inside of each finger and the palm. This glove further records the finger movement. The vibrational frequency generated from the CyberTouch-II ranges from 0–125 Hz with a total of 6 vibrotactile actuators: one on the inside of each finger, one on the palm. Vibrational amplitude is 1.2 N peak-to-peak at 125 Hz (max). Sensor resolution is 1 degree, sensor repeatability is 3 degrees, and sensor data rate is 90 records/sec.

The experimental AR tool-use training task was implemented in Unity (version 2018.3.8f1) and featured a virtual gripper tool consisting of two parallel legs connected to an elongated stick, and a blue cube as the target object that the participant had to enclose with the legs of the gripper tool (cf., Fig. 2). In addition to the AR environment generated in Unity, participants could see the real surface of the table and their hand. The end of the stick was virtually attached to the hand.

The virtual tool was modelled in Unity in a way that when overlaid with the physical table in physical space, its length equated to about 30 cm in the real reaching space of participants when placed at the starting position in front of the participant. Given a forearm length of 25 cm (flat on the physical table), all cubes in the virtual space could be reached. This estimate is not perfectly precise because the apparent size of the entire scene was influenced by the exact distance of the projection screen to the eyes of the participant, which in turn could be modulated by the tightness of fit for the device. However, these differences were minimal. Furthermore, the relative proportions of the virtual scene (including the tool, the plane and the objects therein) were fixed and thus equally affected by any such (small) variations.

Participants performed two blocks of training, one block with visual and vibro-tactile feedback (VT condition) and one block with only visual feedback (V condition). Each block consisted of 120 trials in two half blocks and the order of blocks was randomised among participants.

During training, to start a trial, participants first had to place their hand at a central starting position before them, as indicated by a red square. Distance to the red square was kept constant. The blue target cube then appeared at different locations in the plane in front of the participants. Participants had to move their hand, and thus, the virtual gripper, towards the object to grasp it. The task was to enclose the virtual object with the gripper without touching either side or moving the gripper into the object. In the condition with tactile feedback, touching the object resulted in vibratory feedback to either the thumb (touched left), the index finger (touched right), or to the palm (touched at front). A trial ended when the object was correctly enclosed by the gripper within 20 s and the participant moved the hand back to the start position for the next trial. Participants were informed in advance that an error would occur if they touched (or moved) inside the cube for more than 2 s or if they touched the cube with the tool’s left or right sides for more than 2 s. Then the trial would fail and end.

After 10 consecutive trials, there were 10 s of rest. After each half block of 60 trials, there was one minute of rest. After the first block (i.e., in the middle of the experiment), approximately 10 min rest were granted. Before each condition started and during the 1-min break after the first 60 trials, participants were alerted about the type of feedback.

Before training, participants performed 20 practice trials to learn how to control the virtual tool by moving their right hand, forwards, backwards, left, or right.

The size of each side of the target cube was 40 × 40 mm^2^. Each training half block of 60 trials started with maximally open gripper jaws (120% of the cube width, i.e., 48 mm^3^). Gripper size was then changed adaptively by decreasing the width of the tool in steps of 0.4 mm in a 3 down / 1 up staircase procedure. This is to approach a stable 79.4% correct performance level over the practice trials (Leek 2011). Gripper size at this performance level thus is directly related to the practice effect (PE) in respective half blocks. Minimum gripper size was 40.4 mm. Each participant performed the same number of trials independently of correct or incorrect trials. The total PE was calculated per block as the relative gripper size at the end of the block compared to the starting gripper size. PE was calculated by subtracting 48 mm^3^ as the starting size from the average value of the last 5 trials divided by 48 mm^3^ [(average value − 48)/48]. Therefore, negative values indicate improved performance.

### Measures of ownership and agency

To measure ownership and agency, we adopted the ownership and agency questionnaire by Zhang and Hommel (2015) (cf. Table 1). Each statement was scored on a 7-point Likert scale (-3 “strongly disagree” to + 3 “strongly agree”). Four mean scores were calculated for statistical analysis by aggregating 3 questions each: Q1–Q3 were about the experience of perceiving the hand as one's own hand, i.e., ownership (this variable is abbreviated as BO) and Q7–Q9 were directly associated with the experience of intentional control, i.e., agency (BA). "BO-related" (Q4–Q6) and “BA-related” (Q10–Q12) concerned ownership and agency indirectly (Zhang and Hommel 2015). Scores from Q10–Q12 were reverse-coded, as the corresponding questions are phrased in terms of a loss of control over the tool. According to Kalckert and Ehrsson (2012, 2014), an average score needed to be higher than + 1 to indicate the emergence of ownership and agency. Cronbach’s alpha was calculated for each of the four subscales, with all scales demonstrating acceptable to excellent internal consistency in the first measurement (BO: *α* = 0.87, BO-related: *α* = 0.73, BA: *α* = 0.84, BA-related: *α* = 0.93).

**Table 1:** Statements used in the ownership and agency questionnaire (adapted from Zhang and Hommel 2015)

Variable	Statement
BO	Q1: I felt as if the virtual tool was an extension of my own hand
	Q2: I felt as if the virtual tool was part of my body
	Q3: I felt as if the virtual tool was my hand
BO-related	Q4: It seems as if I had more than one right hand
	Q5: It felt as if my right hand no longer mattered, as if I only needed to sense the virtual tool
	Q6: I felt as if my real hand developed an enhanced sense of virtual touch
BA	Q7: I felt as if I could cause movements of the virtual tool
	Q8: I felt as if I could control movements of the virtual tool
	Q9: The virtual tool was obeying my will and I could make it move just like I wanted it to
BA-related	Q10: I felt as if the virtual tool was controlling my movements
	Q11: It seemed as if the virtual tool had a will of its own
	Q12: I felt as if the virtual tool was controlling me

### Tactile distance judgement (TDJ) task

**Fig. 3:**
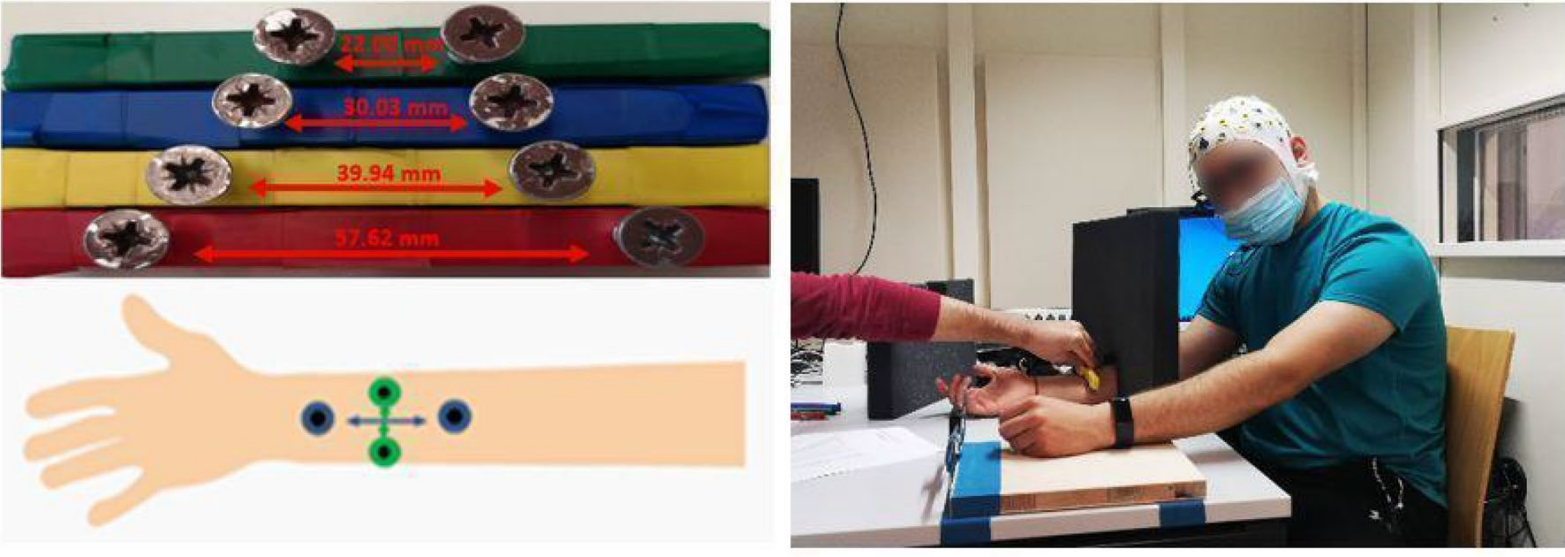
The tactile distance judgement test, with two different orientations (proximodistal, mediolateral) applied to the forearm

We applied a TDJ task as pre-test, mid-test and post-test (adapted from Miller et al. 2014, 2017). Wooden blocks were prepared with 4 sample pairs of screws, each with different distances between them. The screws had round tips with 9 mm diameter. For TDJ testing parallel to the arm axis (“proximodistal” alignment), 3 sample pairs with distances of 57.62, 39.94, and 30.03 mm were prepared. For TDJ orthogonal to the arm axis (“mediolateral”), because of the anisotropy of RFs, the sample with the largest distance (57.62 mm) was replaced by a sample with a smaller distance of 22.00 mm. For each trial, one sample was applied in pseudorandomized order to the right forearm onto the mediolateral or proximodistal orientation (cf., Fig. 3). Each trial lasted approximately 1 s. Each sample was presented 5 times, resulting in 30 trials in total (15 proximodistal and 15 mediolateral; 5 trials each per 3 distances per orientation).

In previous studies (Miller 2014, 2017), participants were instructed to report verbally to the experimenter whether they perceived the distance between the two stimuli on the skin as shorter or longer than a reference in the forehead. In this study, we required participants to report absolute estimates of the distances. This is in accordance with other studies collecting absolute estimates such as verbal estimates (Longo and Golubiova 2017; Fiori and Longo 2018), adjustments of a visually perceived line (Tamè et al. 2021), or kinaesthetic matching of the distance between two fingertips (Keizer et al. 2011; Knight et al. 2014).

Participants were instructed to indicate the perceived distance between the centres of the screws using a digital calliper (analogue scale). The calliper consisted of two steel legs fixed to a piece of wood attached to the table close to the participant’s left hand. The distance between the legs could be easily adjusted by the participant. Right after each TDJ stimulus, participants were asked to use their left hand to report the perceived distance by adjusting the distance between the calliper legs, and the distance was noted by the experimenter based on the mm scale displayed on the calliper monitor (see Fig. 3). Importantly, participants were prevented from seeing the stimuli presented to their forearm by putting an obstacle between their eyes and the right forearm throughout the TDJ test. Therefore, they had no visual information about the real distances, and whether the pairs of stimuli were administered on proximodistal or mediolateral orientation.

The judgement error was used as an indicator of perceived arm length and calculated as the difference between the reported distance and the actual distance that was presented (error = estimated distance—real distance). Positive values thus indicated an overestimation and negative values an underestimation of the distance. Estimation errors were averaged over the five trials per condition (orientation and distance) and calculated separately for pre-test, mid-test and post-test. A decrease in the distance judgment would indicate that after tool-use training, the virtual tool was appended to the sensorimotor representation of the arm within the extent of the existing BS, i.e., the somatotopic cortical representation (cf., Fig. 11). As a consequence, the arm would become perceptually shorter, and different locations on the proximodistal orientation of the forearm would be perceived as closer together.

### Data analysis and statistics

The dataset that was generated and analysed during the current study will be made available on publication in an Open Science Framework repository on OSF.io. Inferential statistics were performed with R (R Core Team 2021) and the Jamovi software environment version 2.2.5.6.2 (The Jamovi project 2021). All relevant R packages (v4.1.1; RStudio v1.4.1717) and related references are listed in the supplement. If not stated differently, *p* < 0.05 were considered as significant, and p values < 0.10 as marginally significant throughout the report.

## Results

### Baseline asymmetry in TDJ error

**Fig. 4:**
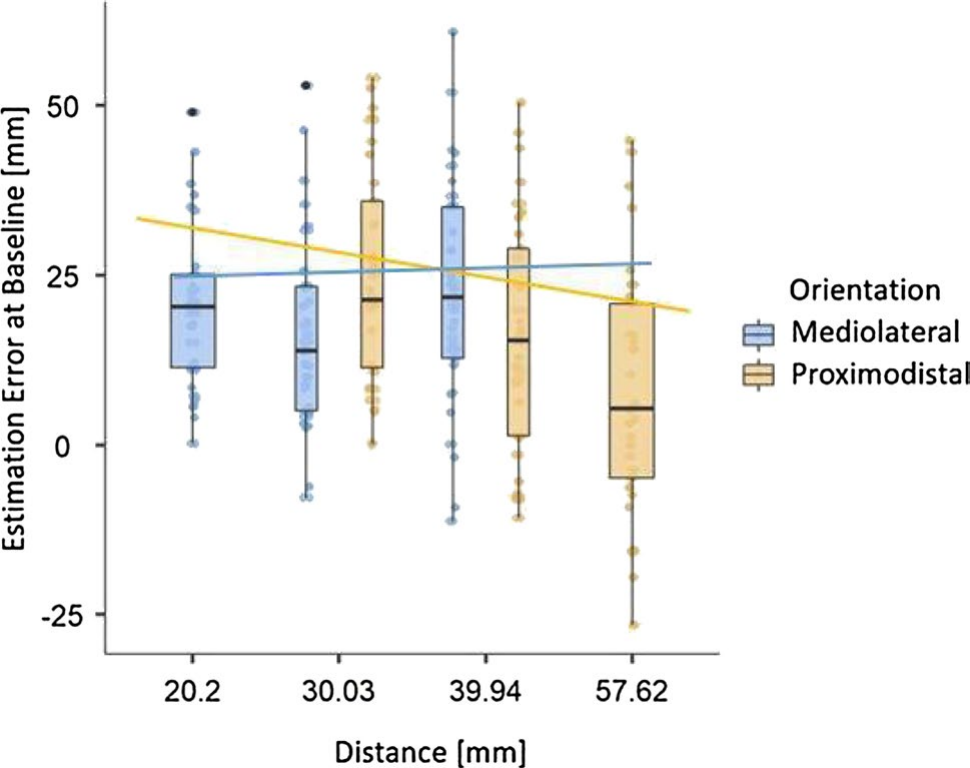
TDJ Estimation Errors in mm at baseline dependent on stimulus Distance and Orientation. Boxes represent medians and interquartile ranges. Whiskers show largest values within 1.5 times the interquartile ranges. Lines show regression lines based on estimates derived from the linear model

First, we examined baseline differences in Estimation Errors for different Orientations and Distances. Figure 4 displays TDJ Estimation Errors at baseline in mm for different Distances and Orientations. For proximodistal, but not mediolateral Orientations, the Estimation Error decreased with increasing distance.

To confirm hypothesised baseline asymmetries of distance judgement errors in the TDJ, we fitted a general linear model (GLM) to predict Estimation Error at baseline with Orientation (proximodistal or mediolateral) and Distance in mm. The model's explanatory power is weak but significant (*R*^2^ = 0.094, adj. *R*^2^ = 0.087, *F*(3,404) = 13.96, *p* < 0.001; cf. Table 2). The model's intercept is at 29.3 (95% CI [23.4, 35.2]), indicating overestimation of tactile distance. Within this model, effects of Orientation (*t*(404) = 3.95, *p* < 0.001) and Distance (*t*(404) = − 3.08, *p* = 0.002), as well as the interaction of Orientation and Distance was significant (*t*(404) = − 3.93, *p* =  < 0.001). Only for the proximodistal orientation, the estimation error decreased with increasing distance.

**Table 2:** Effects of stimulus orientation and distance on estimation errors in the TDJ

	Estimate	*SE*	*t*	*p*
(Intercept)	29.335	2.997	9.79	< .001
Orientation	23.657	5.994	3.95	< .001
Distance	− 0.257	0.083	− 3.08	< .002
Orientation × Distance	− 0.656	0.166	− 3.93	< .001

*df* = 404; Multiple *R*^2^ = .09; Adjusted *R*^2^ = .08; *F*(3, 407) = 13.98; *p* < .001

### Practice effect and role of visual and visuo-tactile feedback

**Fig. 5:**
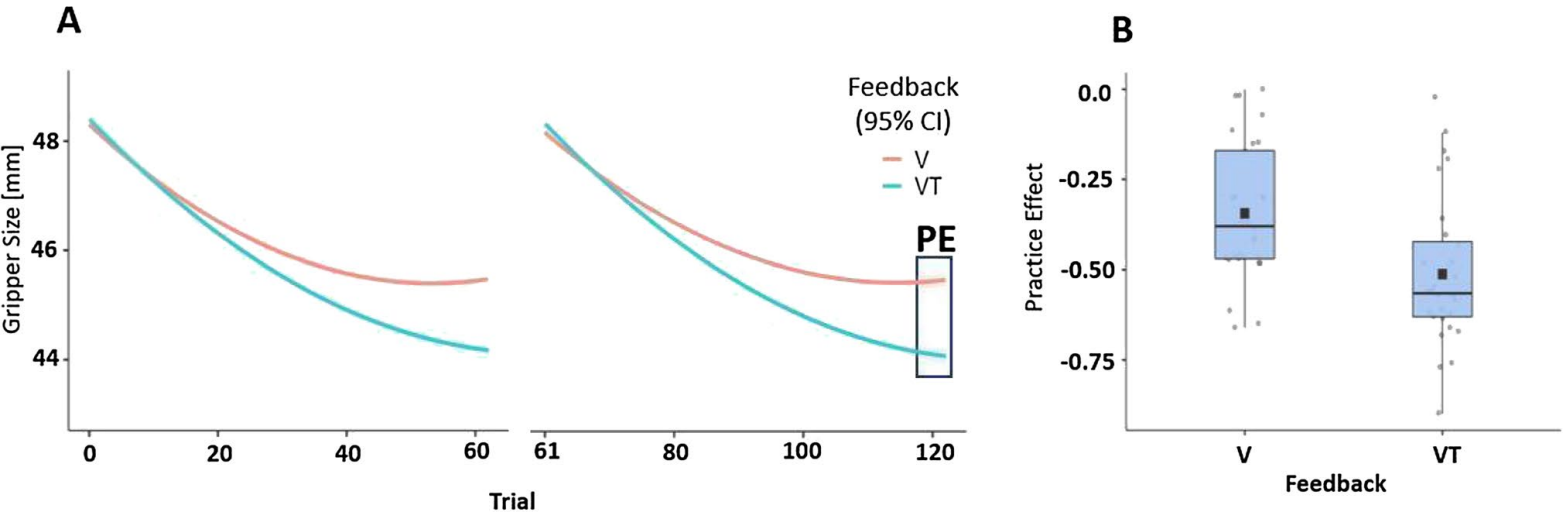
**A** Gripper size during training Blocks with VT versus V Feedback conditions. The square on the right denotes the last 5 trials which were used to calculate the practice effect (PE). **B** Practice effects over the course of the experiment for VT versus V Feedback conditions. Boxes represent medians and interquartile ranges. Whiskers show largest values within 1.5 times the interquartile ranges. Negative values indicate reduced gripper size and better performance

The final performance level as approached by the staircase procedure during training blocks was lower for the VT Feedback compared to the V Feedback condition (cf., Fig. 5). This indicates that VT Feedback was more effective. To quantify and statistically test the effect of Feedback condition on PE, GLM analysis with PE as a dependent variable and Feedback condition (VT or V) and Block number (first or second training block) as factors were conducted. The model’s explanatory power is significant (*R*^2^ = 0.164, adj. *R*^2^ = 0.117, *F*(3, 53) = 3.464, *p* = 0.022; cf., Table 3). The model’s intercept was at − 0.443 (95% CI [− 0.517, − 0.369] indicating a reduction in gripper size on average. The effect of Feedback was significant (*F*(1, 53) = 6.82, *p* = 0.01), but no significant effect of Block nor an interaction effect of Block x Feedback was revealed for PE as a dependent variable.

**Table 3:** GLM for practice effect as dependent variable and Block x Feedback as factors

	SS	df	F	*p*	*η*^2^
Model	0.421	3	3.464	.022	0.164
Block	0.008	1	0.209	.649	0.003
Feedback	0.277	1	6.820	.011	0.108
Block × Feedback	0.014	1	0.344	.56	0.005
Residuals	2.147	53			
Total	2.568	56			

A post hoc t-test revealed that PE was significantly lower (more negative, indicating more reduction in gripper size) for the VT than for the V Feedback condition (*t*(53) =  − 2.611; *p* = 0.01; (cf., Fig. 5).

### Effects of tool-use training on TDJ error

We followed a two-step procedure to test our assumption that training would affect TDJ error in the proximodistal Orientation. We first analysed the effects of distance and baseline error on post-test error. The Residuals of this analysis represent the variance in error after training that is not explained by baseline and distance and was then used for analysing the Orientation effect on TDJ error after training. This allowed us to evaluate Orientation effects on Estimation Errors after training regardless of the different Distances used for both Orientations. Without any training effect, Residuals should be around zero. Negative residuals would indicate reduced Estimation Errors.

**Fig. 6:**
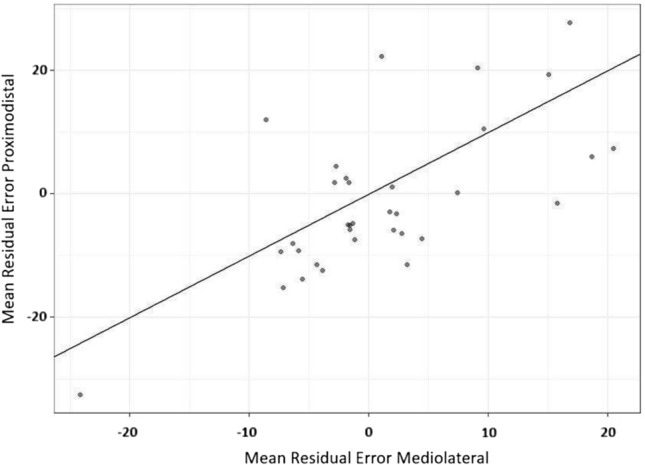
Mean Residual Estimation Errors per individual for proximodistal and mediolateral Orientations. The solid line represents the diagonal. Points below the diagonal indicate Residuals lower for proximodistal than for mediolateral Orientations in the same individual

**Fig. 7:**
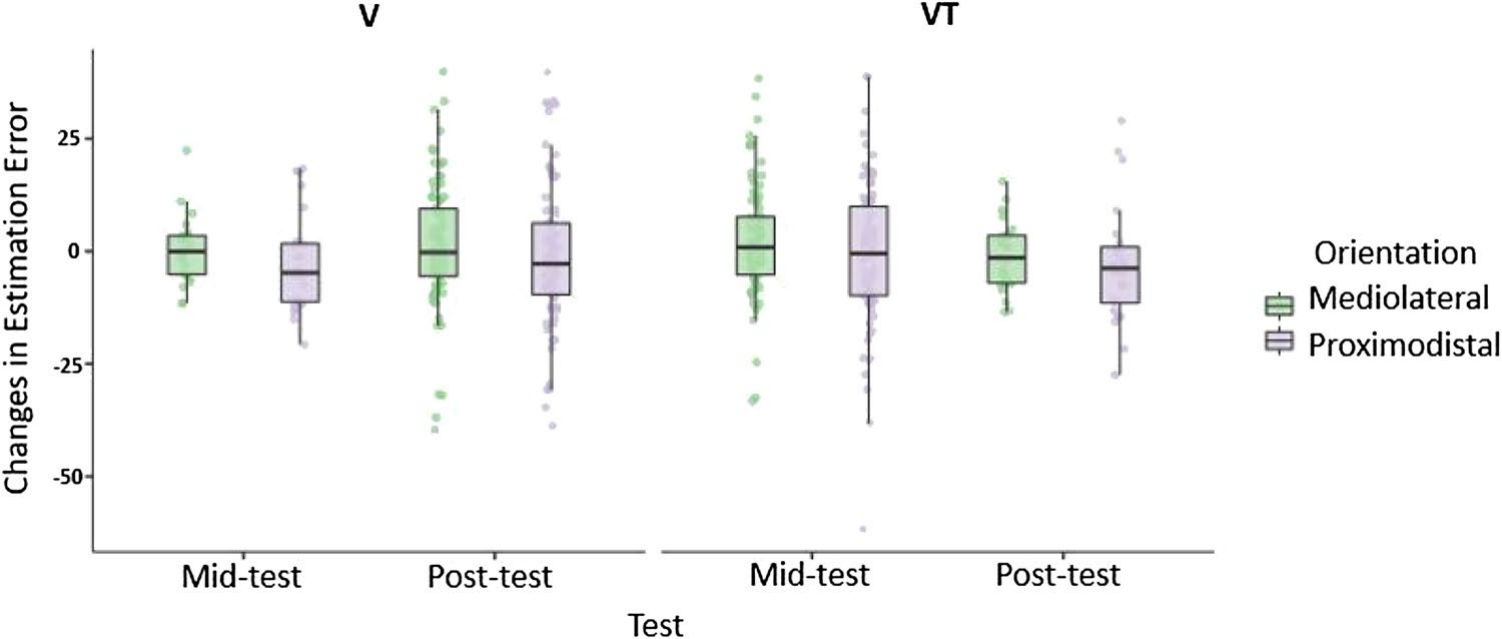
Changes in Estimation Error at mid-test and post-test for mediolateral and proximodistal Orientations and for Feedback conditions. Boxes represent medians and interquartile ranges. Whiskers show largest values within 1.5 times the interquartile ranges

As illustrated in Fig. 6, there was a tendency that average Residuals per individual for proximodistal Orientation were lower than those for mediolateral Orientation. As the assumption of normality was violated (Shapiro–Wilk *W* = 0.93, *p* = 0.028), we performed a one-sided one-sample Wilcoxon Rank test to compare both Orientations. Result revealed that Residuals for the proximodistal Orientation (median = − 4.1, SE = 2.1) were significantly lower and more negative than those for the mediolateral Orientation (median = − 1.3, SE = 1.6; *W*(33) = 416, *p* = 0.043). Therefore, one might conclude that perceived arm length (proximodistal Orientation) but not arm width (mediolateral Orientation) was reduced (cf., Fig. 7).

However, Orientation effects on the residuals were not very robust and disappeared when taking Feedback and Test (mid-test, post-test) into account. We fitted a linear model to predict the Residual Estimation Error with Orientation, Test and Feedback. The model’s explanatory power is very weak and not significant (*R*^2^ = 0.01, adj. *R*^2^ = − 0.004, *F*(7, 407) = 0.77, *p* = 0.607*;* cf., Table 4). The model’s intercept is at − 0.56 (95% CI [− 2.17, 1.04]). Within this model, no significant main or interaction effects could be revealed.

**Table 4:** Effects of Orientation, Test and Feedback on changes in Residual Estimation Error after training

	Estimate	*SE*	*t*	*p*
(Intercept)	− 0.564	0.818	− 0.69	< .491
Orientation	− 2.536	1.637	1.55	.122
Test	− 0.791	1.637	0.48	.629
Feedback	− 0.069	1.637	− 0.04	.966
Orientation × Test	0.789	3.273	0.24	.810
Orientation × Feedback	− 0.026	3.273	− 0.008	.994
Test × Feedback	− 4.262	3.273	− 1.3	.194
Orientation × Test × Feedback	0.063	6.547	0.009	.992

*df* = 407; Multiple *R*^2^ = .01; Adjusted *R*^2^ = − .004; *F*(7, 407) = .77; *p* = .607

### Body ownership and body agency after virtual tool-use

**Fig. 8:**
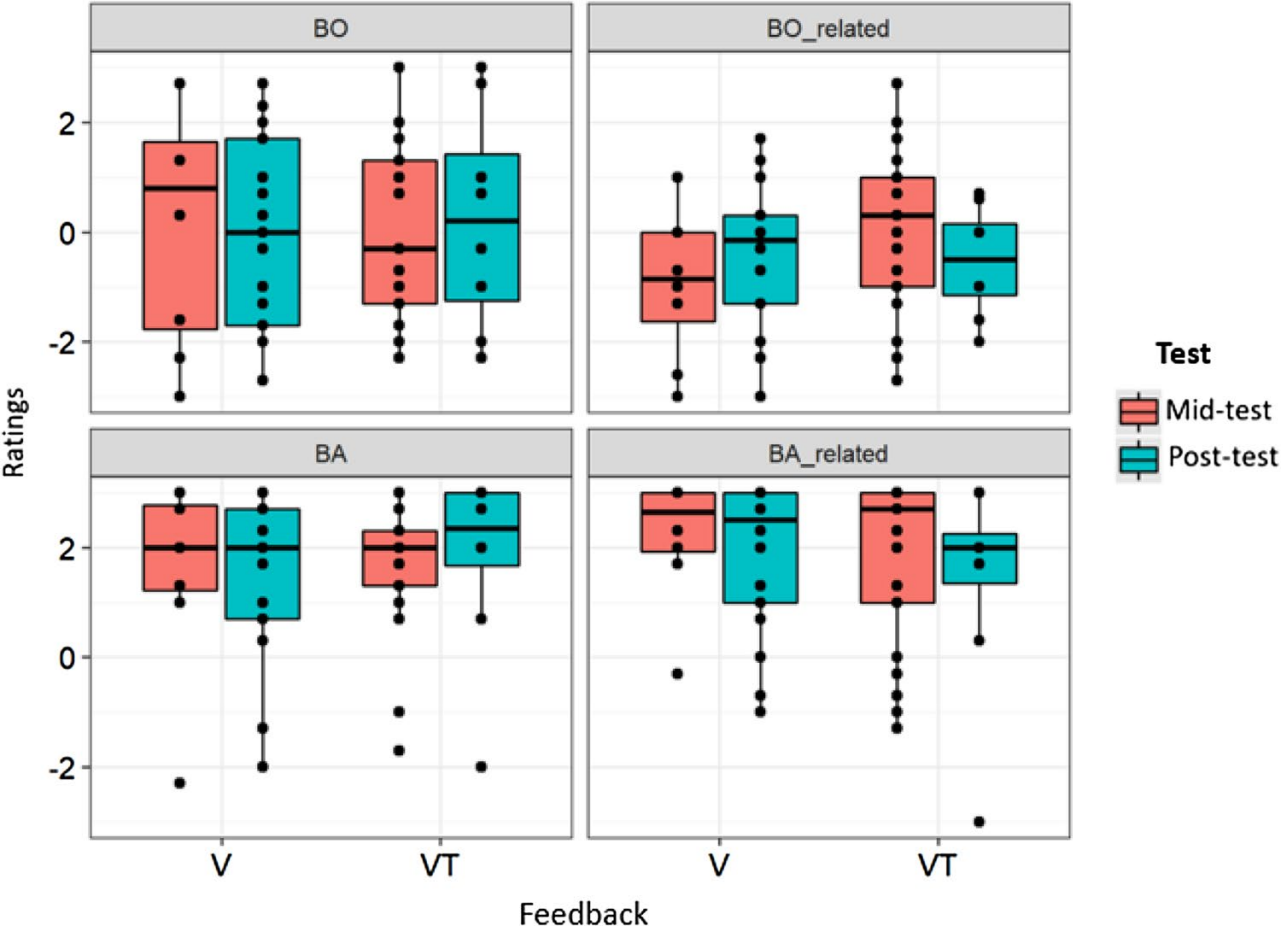
Ratings of BO, BO-related, BA, and BA-related subscales depending on Feedback condition and Test. Boxes represent medians and interquartile ranges. Whiskers show largest values within 1.5 times the interquartile ranges

Descriptive analysis of ownership and agency revealed values of 0.0 ± 0.1 and − 0.3 ± 0.1 for BO and BO-related, and 1.7 ± 0.1 and 1.8 ± 0.1 for BA and BA-related, respectively (means and *SE*). Thus, only mean values for BA and BA-related, but not BO and BO-related, were above 1 and thus above the threshold suggested by Kalckert and Ehrsson (2012, 2014). Figure 8 illustrates these findings separated by Feedback condition and Test.

To analyse whether emergence of ownership and agency was dependent on Feedback, Test, BS plasticity or PE, we performed a three-step linear regression separately for ownership and agency ratings. In the first step, we analysed whether ratings were predicted by Feedback and Test or the interaction of Feedback and Test (model 1). In the second step we added PE (model 2), and in the third step we added TDJ Estimation Error to the regression model (model 3; cf. Table 5).

**Table 5:** Model fit parameters of the three linear regression models for Ownership and Agency variables

	Model 1						Model 2						Model 3					
	*R*	*R*^2^	Adjusted *R*^2^	*F*	RMSE	*p*	*R*	*R*^2^	Adjusted *R*^2^	*F*	RMSE	*p*	*R*	*R*^2^	Adjusted *R*^2^	*F*	RMSE	*p*
BO	0.23	0.05	0.002	1.05	1.67	.379	0.33	0.11	0.04	1.64	1.62	.177	0.41	0.16	0.08	2.05	1.57	.087^**+**^
BO-related	0.23	0.05	0.0007	1.01	1.30	.394	0.31	0.09	0.03	1.43	1.26	.23	0.43	0.18	0.11	2.36	1.20	.052^**+**^
BA	0.08	0.006	− 0.05	0.12	1.38	.948	0.21	0.04	− 0.03	0.58	1.35	.676	0.23	0.05	− 0.04	0.58	1.34	.710
BA-related	0.24	0.06	0.004	1.08	1.32	.362	0.24	0.06	− 0.014	0.81	1.32	.528	0.24	0.06	− 0.03	0.63	1.32	.675

***p < .001, **p < .01, *p < .05,  ^+^ *p* < .10

For BO, model 1 was not significant (*R*^2^ = 0.05, adj. *R*^2^ = 0.002; *F*(3, 53) = 1.05, *p* = 0.378; cf. Table 5). Adding PE (model 2) slightly improved the initial model (*p* = 0.075; cf. Table 6), but it still remained not significant (*R*^2^ = 0.11, adj. *R*^2^ = 0.04; *F*(4, 52) = 1.64, *p* = 0.177; cf. Table 5). Adding Residual Estimation Error (model 3) again slightly improved the initial model (*p* = *0.073;* cf., Table 6), and this model was also marginally significant (*R*^2^ = 0.16, adj. *R*^2^ = 0.08; *F*(5, 51) = 2.05, *p* = 0.087; Table 5). Further, it revealed a marginally significant prediction effect of PE (beta = − *2.153, SE* = *1.132; p* = 0.063; Table 7) and a marginally significant prediction effect of Residual Estimation Error for BO (beta = − *0.03, SE* = *0.016; p* = 0.073; cf., Table 7). BO ratings increased with increasing performance level (decreasing PE) and decreasing Estimation Error, i.e., more BS plasticity (cf., Fig. 10). No effects of Test or Feedback or their interaction could be revealed.

**Table 6:** Model comparison of the three linear regression models for Ownership and Agency variables

Comparison	BO			BO-related			BA			BA-related		
	Δ*R*^2^	*F*	*P*	Δ*R*^2^	*F*	*P*	Δ*R*^2^	*F*	*P*	Δ*R*^2^	*F*	*P*
Model 1 vs. 2	0.05	3.30	.075^+^	0.04	2.58	.114	0.03	1.96	0.167	2.87	0.01	.901
Model 2 vs. 3	0.05	3.35	.073^+^	0.08	5.60	.022*	0.01	0.61	0.436	1.13	0.006	.938

***p < .001, **p < .01, *p < .05,  ^+^ *p* < .10

**Table 7:** Linear regression for Ownership and Agency variables as dependent variables, Feedback and Test as factors and Residual Estimation Error and Practice Effect as covariates

Independent variables	BO			BO-related			BA			BA-related		
	Model 1	Model 2	Model 3	Model 1	Model 2	Model 3	Model 1	Model 2	Model 3	Model 1	Model 2	Model 3
Intercept	0.940 (0.775)	0.200 (0.861)	0.140 (0.843)	− 1.120 (0.601)	− 1.631** (0.672)	− 1.69** (0.644)	1.340* (0.638)	0.863 (0.718)	0.885 (0.721)	2.540*** (0.610)	2.498*** (0.699)	2.496*** (0.706)
Feedback	− 1.015 (0.852)	− 1.328 (0.852)	− 1.319 (0.833)	1.058 (0.661)	0.841 (0.665)	0.850 (0.637)	0.364 (0.702)	0.163 (0.710)	0.160 (0.712)	− 0.523 (0.671)	− 0.541 (0.692)	− 0.540 (0.698)
Test	− 1.065 (0.852)	− 1.045 (0.834)	− 1.015 (0.815)	0.65 (0.661)	0.633 (0.651)	0.692 (0.624)	0.314 (0.702)	0.327 (0.696)	0.316 (0.698)	− 0.523 (0.671)	− 0.522 (0.677)	− 0.521 (0.684)
Feedback x Test	2.240* (1.265)	2.060 (1.243)	2.017 (1.215)	− 1.162 (0.981)	− 1.287 (0.970)	− 1.327 (0.930)	− 0.593 (1.043)	− 0.710 (1.037)	− 0.694 (1.040)	− 0.568 (0.997)	− 0.579 (1.009)	− 0.579 (1.019)
PE		− 2.102 (1.158)	− 2.153^**+**^ (1.132)		− 1.451 (0.904)	− 1.502^**+**^ (0.866)		− 1.354 (0.966)	− 1.335 (0.969)		− 0.118 (0.940)	− 0.119 (0.949)
Residual Estimation Error			− 0.03^**+**^ (0.016)			− 0.288** (0.012)			0.0107 (0.013)			− 0.001 (0.013)

Beta Coefficients and Standard errors (in parenthesis) are reported. ***p < .001, **p < .01, *p < .05, ^+^*p* < .10

**Fig. 9:**
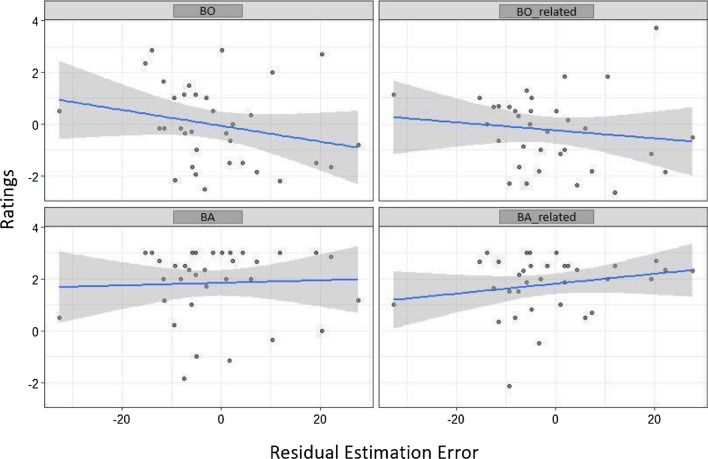
Associations between Residual Estimation Error for proximodistal Orientations in mm and Ownership and Agency ratings. Solid lines and shaded areas represent linear regression lines and 95% confidence intervals

**Fig. 10:**
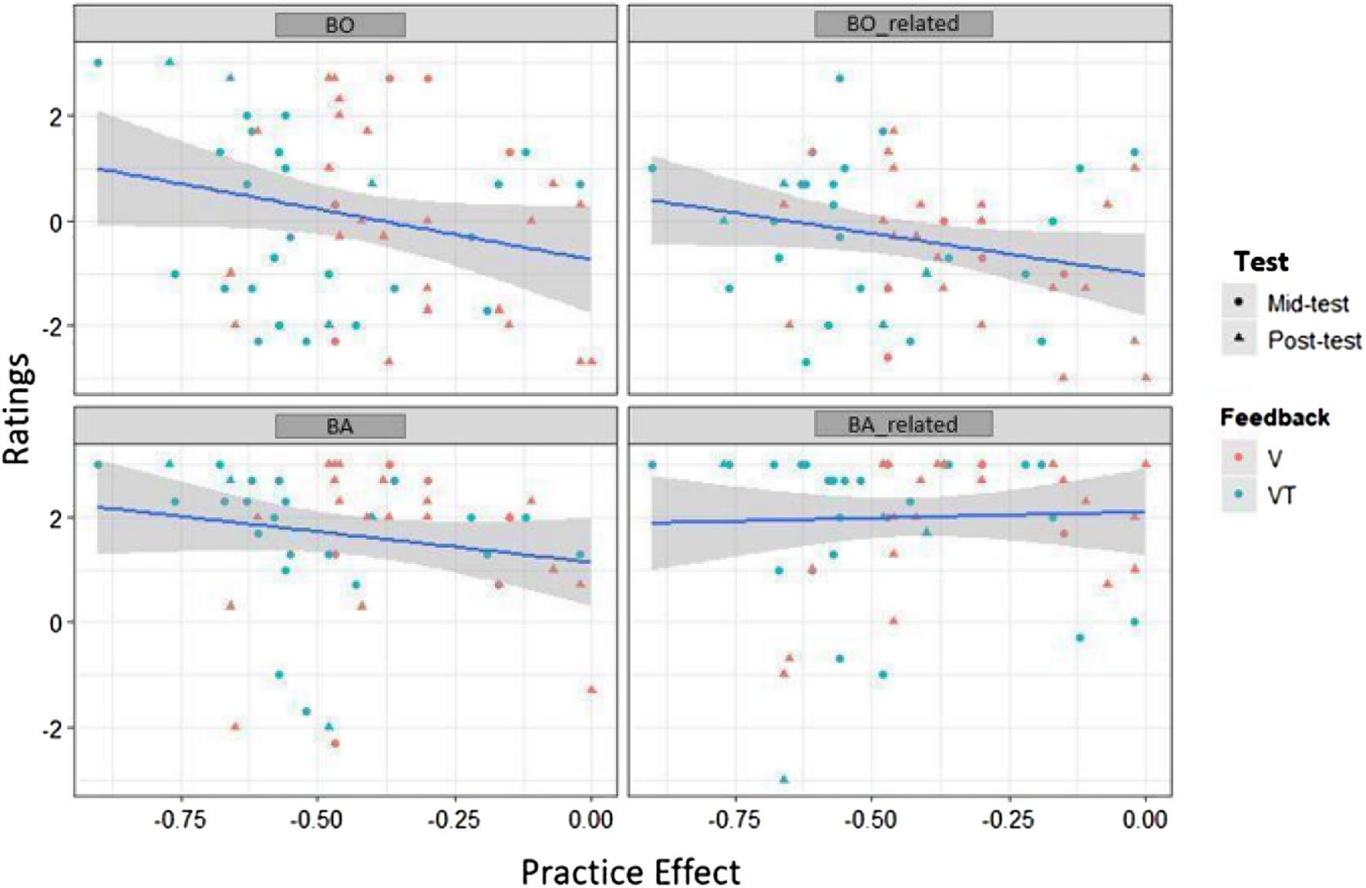
Associations between Practice Effect during virtual tool-use training and Ownership and Agency ratings dependent on Test and Feedback. Solid lines and shaded areas represent linear regression lines and 95% confidence intervals

For BO-related, model 1 was not significant (*R*^2^ = 0.05, adj. *R*^2^ = 0.0007; *F*(3, 53) = 1.01, *p* = 0.394). Adding PE (model 2) did not improve the initial model (*p* = 0.114; cf., Table 6) and the model was still not significant (*R*^2^ = 0.09, adj. *R*^2^ = 0.03; *F*(4, 52) = 1.43, *p* = 0.237; cf., Table 5). Adding Residual Estimation Error in model 3 significantly improved the model (*p* = 0.022; cf., Table 6) and the model became marginally significant (*R*^2^ = 0.18, adj. *R*^2^ = 0.11; *F*(5, 51) = 2.36, *p* = 0.052; cf., Table 5). Model 3 revealed a significant prediction of BO-related by PE (beta = − 1.502, SE = 0.866; *p* = 0.089; cf., Table 7) and Residual Estimation Error (beta = − 0.288, SE = 0.012, *p* = 0.022; cf. Table 7). As for BO, BO-related ratings increased with decreasing Estimation Error, i.e., more BS plasticity (cf., Fig. 9) but also with increased performance level (more negative PE; cf., Fig. 10). Again, no effects of Test or Feedback or their interaction could be revealed.

For BA, model 1 was not significant (*R*^2^ = 0.006, adj. *R*^2^ = − 0.05; *F*(3, 53) = 0.12, *p* = 0.948: cf., Table 5). Adding PE in model 2 (beta = − 1.354, SE = 0.966, *p* = 0.167; cf., Tables 6 and 7) did not improve the model significantly (*R*^2^ = 0.04, adj. *R*^2^ = − 0.03; *F*(4, 52) = 0.58, *p* = 0.676; Tables 5). Further adding Residual Estimation Error in model 3 (beta = − 0.010, SE = 0.013, *p* = 0.436; cf., Tables 6 and 7) also did not improve the initial model (*R*^2^ = 0.05, adj. *R*^2^ = − 0.04; *F*(5, 51) = 0.58, *p* = 0.710; cf., Table 5). Thus, neither PE nor BS plasticity predicted agency ratings (cf., Figs. 9 and 10). Also, neither effects of Test nor Feedback or their interaction could be revealed.

For BA-related, model 1 was not significant (*R*^2^ = 0.06, adj. *R*^2^ = 0.004; *F*(3, 53) = 1.08, *p* = 0.362; cf., Table 5). Again, adding PE (model 2; *R*^2^ = 0.06, adj. *R*^2^ = − 0.01; *F*(4, 52) = 0.81, *p* = 0.528; beta = − 0.118, SE = 0.940; *p* = 0.901; cf., Tables 5 and 7) and Estimation Error (model 3; *R*^2^ = 0.06, adj. *R*^2^ = − 0.03; *F*(5, 51) = 0.63; *p* = 0.675, beta = − 0.001, SE = 0.013, *p* = 0.938; cf., Tables 5 and 7) did not improve the initial model (*p* = 0.901 and *p* = 0.938, respectively; cf., Table 6). Thus, neither PE nor BS plasticity predicted agency-related ratings (cf., Fig. 9). Again, neither effect of Test or Feedback or their interaction could be revealed.

## Discussion

In this study, we aimed at better understanding the emergence of perceived sense of body ownership and sense of agency during virtual tool-use training with different types of feedback and its dependency on BS plasticity as revealed by changes in tactile distance estimation on the arm.

### Virtual tool-use training induces changes in the body schema

We cannot support findings of Baseline asymmetries in distance judgement errors between proximodistal and mediolateral orientations have been previously reported (Miller et al. 2016; Knight et al. 2014). Such asymmetries, with distances being perceived as smaller for proximodistal than for mediolateral orientations, could be suggested based on the oval shape of RFs, which are larger along the proximodistal compared to the mediolateral body axis (Longo 2020; Mainka et al. 2021). On the contrary to these findings, in the current study, estimation errors became smaller in the proximodistal orientation but only for larger distances that were not measured for the mediolateral orientation. However, this result must be taken with care because the explanatory power of the model was weak, and the results might be biased by the use of different distances in the proximodistal and mediolateral orientations.

**Fig. 11:**
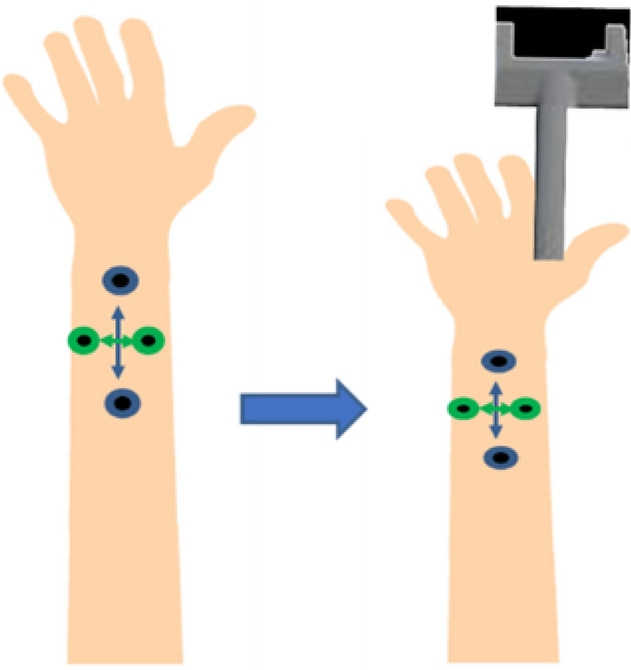
Schematic illustrating the incorporation of the tool into the BS

Virtual tool-use training resulted in smaller residual estimation errors, i.e., reduced overestimation of distances in the proximodistal orientation, which is in line with previous studies in non-virtual settings (e.g., Iriki et al. 1996, 2011; Cardinali et al. 2009a, b; Sposito et al. 2012; Miller et al. 2014, 2017). While it was previously suggested that the incorporation of the tool into the existing BS leads to an increase of the perceived arm length (for review, e.g., Sposito et al. 2012), and consequently to a shift of the perceived boundary of reachable space (Maravita and Iriki 2004), our results are consistent with the notion that after tool-use training, the virtual tool was appended to the sensorimotor representation of the arm within the extent of the existing BS, i.e. the somatotopic cortical representation (cf., Fig. 11). Thus, in our study the arm became perceptually shorter, and different locations on the proximodistal orientation of the forearm were perceived as closer together. Given that there is a close association between BI and BS, i.e., the conscious BI will depend on the information provided by the unconscious BS, a consciously perceived reduction of the arm length (= BI) could be caused by an unconscious alteration of the somatotopic body map in the somatosensory cortex (= BS), where a reduced representation of the arm is seen due to incorporation of the tool within the given space of the map. Under different experimental conditions, it might be that participants project their own hand/arm to the external position of the (virtual) tool like in body ownership illusions. Here, the BS might be unchanged but differently integrated with the coordinates of the external space, leading to perceived enlargement of the own body in the BI. This could explain contradictory findings in previous studies. Martel et al. (2021, 2019) reported a similar reduction of consciously estimated arm length after tool use under vision, but not when blindfolded. One might speculate that a shift of visual spatial attentional focus to the (end of) the tool facilitates BS plasticity and perceived shrinkage of the own arm (cf., Rossetti et al. 2015).

Interestingly, Canzoneri et al. (2013) observed that after tool-use training, subjects not only perceived distances between two stimuli as smaller in the proximodistal but also as larger in the mediolateral direction in a tactile distance perception task administered on their forearm. We speculate that this might be an effect of the audio-tactile interaction task employed by Canzoneri et al. (2013), in which participants were blindfolded and exposed to auditory stimulation (pink noise) which potentially restricted their peripersonal space and narrowed their BI. In contrast, in our own and other previous work (e.g., Miller et al. 2014, 2017), the visual domain is likely to have made a strong contribution to learning during the tool-use training. Here, future experiments with the aim to leverage AR and audio-tactile stimulation could help resolve and better understand this apparent discrepancy.

Additionally, we further examined whether there was a practice effect during the training and if this effect was dependent on the type of feedback (visuo-tactile or visual only) given during each block. As expected, participants learned virtual tool-use better during training with visuo-tactile feedback, resulting in smaller gripper size at the end of the practice block. Also, a steeper learning slope was observed during this type of feedback compared with vision-only feedback. This highlights the importance of multisensory integration in tool-use learning. However, since immersive AR provides an environment in which different sensory information channels can be isolated, we suggest future studies to examine in detail the contribution of each sensory modality to the learning effect.

### Changes in ownership but not agency relate to altered body schema and  practice effect

Participants developed a modified sense of agency during training, but no association with PE or BS plasticity was revealed. However, ratings were very high in all conditions and a ceiling effect might have masked potential effects. Although, on average, participants did not develop a sense of body ownership over the tool, regression analyses indicated that on individual level there were negative associations between estimation error and ownership ratings. Thus, even with the average effect of tool-use training on the BS being not very robust, the more the overestimation in the TDJ as a sensitive measure of representational plasticity along the proximodistal orientation was reduced, the higher the ownership ratings became. This suggests that plastic reorganisation of the sensorimotor representation, i.e., the BS, was indeed associated with an increase in the subjective experience of the tool being a part of one's body. Thus, our findings suggest that the emergence of a sense of body ownership may strongly relate to changes in the sensorimotor BS, which then are reflected in an altered BI. Additionally, ownership ratings were higher with larger practice effects.

Cardinali et al. (2021) and Rossetti et al. (2015) used skin conduction measurements to investigate embodiment, i.e., changes in body ownership over the used tool. In the study by Cardinali et al. (2021), body ownership over a mechanical grabber (arm-shaped tool) was expressed in an altered skin conductance response to a threatening stimulus approaching the tool. This was interpreted as that the tool was perceived as part of the own body. Rossetti et al. (2015) found that tool-use training expanded the space around the body in which an approaching stimulus was perceived as a thread as indicated by induced changes in skin conductance. These findings were interpreted as an enlargement of the peripersonal space through the incorporated tool. Recently, Miller et al. (2014, 2017), reported that illusory embodiment is dependent on the shape of the tool. While an arm-shaped tool modulated tactile perception on the arm but not the hand, a hand-like tool altered perception on the hand but not the arm. Extending these findings, Cardinali et al. (2021) reported that not only visual similarity but also similarity in potential actions performed with the tool or the own hand is an important factor facilitating the illusory embodiment. Tool-use in virtual or augmented realities might be an ideal solution to experimentally manipulate function and morphology of the tool to investigate their effects on tool embodiment and associated alterations in BS and BI, as these multilayer phenomena cannot be captured in a single task (Cardinali et al. 2021).

### Manipulating feedback conditions in VR and AR

We found steeper learning curves and stronger PE for training with visuo-tactile feedback as compared to visual feedback alone. This is in line with the notion that tactile feedback plays an important role in sensorimotor control of grasping and manipulation of objects (D’Alonzo et al. 2015). D’Angelo et al. (2018) trained participants to control a virtual hand in VR to grasp an object and examined the sensorimotor representation of forearm length using a forearm bisection task and a reaching distance task. Their results suggested that synchronous movements of real and virtual hands may lead to changes in the forearm BS, which is again consistent with the argument that multisensory, i.e., visual and proprioceptive, feedback may play a critical role in mediating this effect. When the hand interacts with the physical object in daily life, sensorimotor information of mechanical contact is necessary to enable planning and controlling of the object manipulation (Johansson and Flanagan, 2009) and vision-only input is not sufficient for “correction and control of grasping and manipulation” (Stephens-Fripp et al. 2018).

Contrary to our expectation, the type of feedback did not play a role in the association between altered BS and emergence of ownership and agency. Therefore, our results are not consistent with previous studies which reported that touch, nociception, proprioception, and tactile feedback were essential for embodiment (Beckerle et al. 2018; Azañón et al. 2016), and that a high degree of BR malleability during action performance was linked to both tactile and visual feedback. One might speculate that when touching a physical object in real life we do not receive a vibration and the sense of touch is different. Thus, it might be that synchrony is not as precise as with simple touch.

Ownership for a virtual hand/tool could be modulated and enhanced through sensory feedback and multisensory integration (Beckerle et al. 2018), wherein an interaction of synchronous visuo-tactile feedback had been argued to lead to an enhanced sense of body ownership (Richard et al. 2021). Interestingly, in the case of conflicting visual and tactile information, the visual information seemed to be a primary factor in the embodiment process. We assume that, although BS plasticity also was independent of feedback conditions on average, on individual level combined visual and tactile feedback might have facilitated BS plasticity and, as a consequence, emergence of ownership.

## Conclusions and outlook

The present study addressed some important methodological gaps in the literature, demonstrating systematic malleability of the sensorimotor BS in a new experimentally controlled and ecologically valid paradigm for virtual tool-use training. Overall, our findings suggest that virtual tools can be incorporated into the existing BS of the forearm, while showcasing how future work may further disambiguate the contributions of tactile and visual feedback. At present, it appears that the visual domain may have a leading role in this process. However, our present work is still limited because the visual modality was always available and could not be controlled in any way. This might be addressed in future work that presents tactile feedback with and without visual feedback.

In addition, our self-report results suggest that the AR tool may be perceived as sufficiently similar to a physical tool that moves directly with own’s hand. Our results are further consistent with the notion that the tool-use dependent representational plasticity of a body part and the specificity corresponds with the shape of the tool (Miller et al. 2014). In other words, our findings indicated transferability of the BS representational plasticity induced by an arm-shaped virtual tool towards real arm representations.

Finally, the present work suggests that vibro-tactile and visual feedback may jointly increase tool embodiment in AR, although further studies with similar paradigms are needed. We believe that this may have implications for further neurobehavioral work in experimental psychology, as well as with respect to designing more immersive and realistic tool-use experiences in other AR application contexts. For example, further work on this intersection could contribute to our understanding of challenging real-world use cases, such as requirements for BS malleability in the context of virtually operating theatres (see, e.g., Zaman et al. 2019). Together, our findings thus substantially extend previous studies by demonstrating how even virtual tools become incorporated into the BS, while adding to our understanding of the respective roles of visual and haptic feedback in virtual tool-use training in general.

## Supplementary Information

Below is the link to the electronic supplementary material.

Supplementary file1 (DOCX 19 KB)

## Data Availability

The dataset that was generated and analysed during the current study will be made available on publication in an Open Science Framework repository on OSF.io.
